# Improving eDNA yield and inhibitor reduction through increased water volumes and multi-filter isolation techniques

**DOI:** 10.1038/s41598-019-40977-w

**Published:** 2019-03-27

**Authors:** Margaret E. Hunter, Jason A. Ferrante, Gaia Meigs-Friend, Amelia Ulmer

**Affiliations:** 1 U.S. Geological Survey, Wetland and Aquatic Research Center, 7920 North West 71st Street, Gainesville, Florida 32653 USA; 2 Cherokee Nation Technologies, contracted to the U.S. Geological Survey, Wetland and Aquatic Research Center, 7920 North West 71st Street, Gainesville, Florida 32653 USA

## Abstract

To inform management and conservation decisions, environmental DNA (eDNA) methods are used to detect genetic material shed into the water by imperiled and invasive species. Methodological enhancements are needed to reduce filter clogging, PCR inhibition, and false-negative detections when eDNA is at low concentrations. In the first of three simple experiments, we sought to ameliorate filter clogging from particulates and organic material through a scaled-up, multi-filter protocol. We combined four filters in a 5 mL Phenol-Chloroform-Isoamyl (PCI) procedure to allow for larger volumes of water (~1 L) to be filtered rapidly. Increasing the filtered water volume by four times resulted in 4.4X the yield of target DNA. Next, inhibition from organic material can reduce or block eDNA detections in PCR-based assays. To remove inhibitory compounds retained during eDNA isolation, we tested three methods to chemically strip inhibitors from eDNA molecules. The use of CTAB as a short-term (5–8 day) storage buffer, followed by a PCI isolation, resulted in the highest eDNA yields. Finally, as opposed to a linear relationship among increasing concentrations of filtered genomic eDNA, we observed a sharp change between the lower (70–280 ng) and higher (420–560 ng) amounts. This may be important for effectively precipitating eDNA during protocol testing.

## Introduction

Environmental DNA (eDNA) originates from cellular material shed by organisms (via skin, excrement, saliva etc.) into natural substrates such as water, air or soil, and can be used for non-invasive detection of species for conservation and management actions^1–5^. Environmental DNA methods can be used to define species range limits, elucidate occurrence and detection estimates, and identify movement corridors or invasion pathways of non-native species^5–11^. However, in some systems, challenges remain in both recovering sufficient eDNA yields and filtering enough water volume for accurate detection. Low concentrations can result when target animals are small or have limited abundance, or when species shed or excrete eDNA at low rates, as with semi-aquatic reptiles^12,13^. Further, detection of low quantity eDNA is particularly constrained when PCR inhibitors are present in the sample medium^14^.

Several studies have identified the underlying mechanisms surrounding eDNA capture and detection and have proposed protocols to improve eDNA yields^15–19^. Wilcox, *et al*.^15^ found that most eDNA is cell-bound and larger filter sizes (~1.2 μm) are sufficient to capture the cells while simultaneously allowing particulates in the water to pass through. However, even with larger filters, clogging can still occur^11,20,21^. Longmire’s lysis buffer has been identified as the optimal buffer for long-term storage and maximum recovery of eDNA yields^22^. Additionally, PCR inhibitors have been reduced through sample dilution or physically removed using inhibitor removal kit (IRK) columns^14^. We found that applying these various techniques to sample our ecological system did not sufficiently reduce PCR inhibitors or filter clogging, nor did it increase eDNA yields. Therefore, we conducted three simple experiments to improve yield and inhibitor removal in eDNA samples, and to improve method and protocol development utilizing genomic DNA (gDNA)^17,19^. These independent experiments resulted in (1) improved eDNA yields through the use of larger water volumes and multiple filters, (2) optimized storage buffer and isolation methods to reduce PCR inhibitors, and (3) evidence that increasing concentrations of filtered eDNA do not result in linear yields of isolate.

The volume required for filtration is generally based on the concentration of target species eDNA in the water sample, i.e., the lower the concentration of target eDNA, the more water needed for detection. Filtration volumes in the literature vary from 15 mls to 6 L and higher (see^23,24^); however, the filtration of large water volumes is often difficult due to sediment and other biological particulates (e.g., algae, microorganisms) clogging individual filters. Therefore, to improve eDNA detection we focused our first experiment on filtering larger water volumes by scaling-up the typical one filter isolation protocol to isolate multiple filters collectively.

In our second experiment, we focused on reducing PCR inhibitors bound to eDNA molecules^4,14,25^. In some ecosystems, such as the tea-colored waters of the southern U.S. rivers and estuaries, particularly high levels of PCR inhibitors are found^14,26^. The color is typically caused by decaying organic material, such as tannins from pines and mangroves, and humic acid from soils. Tannic and humic acids can inhibit PCR amplification through mechanisms including reducing DNA polymerase activity, changing buffer composition, binding to nucleic acids, and/or by quenching the fluorescence signal of the DNA binding dyes used in quantitative PCR assays^27–29^. We apply IRK columns to remove inhibitors without diluting low concentration eDNA post isolation^14^. However, heavily inhibited water requires numerous IRK columns to allow for proper assay efficiency and detection, and DNA can be lost in this extended process. We attempted to improve PCR inhibitor removal through chemical separation during the isolation process. Longmire’s buffer and Cetyl trimethylammonium bromide (CTAB) are traditional lysis buffers to store and preserve DNA^22,30–32^. To remove inhibitors, PCI and CTAB DNA isolation methods are commonly applied^30,33,34^. Therefore, the combination of Longmire’s and PCI are frequently used for eDNA isolations^22,35–37^. We tested three different combinations of these buffers and isolation procedures and used droplet digital PCR (ddPCR) to quantify their relative effectiveness for inhibitor reduction in tannin-laden creek water.

Environmental DNA methods development often utilizes gDNA for testing^14,38–40^ but low concentrations of eDNA could be lost due to insufficient pelleting during isolations^4,25,41^. DNA is also thought to adhere to many plastics, further reducing eDNA yields. In our last experiment, we evaluated whether eDNA concentration measurements increase in a linear fashion with increasing initial amounts of filtered Grass Carp (*Ctenopharyngodon idella*) genomic DNA (gDNA). The information from these three experiments could be used individually, or in combination, to improve the processing and detection of samples containing inhibitory compounds or low eDNA concentrations.

## Results

### Do multiple filter isolations result in greater eDNA yield?

**Figure 1:**
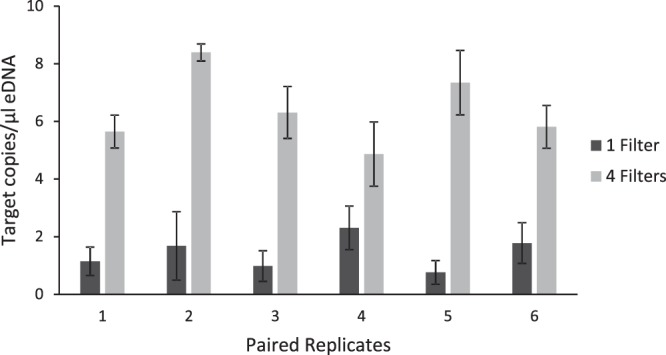
Average concentrations (target copies/µl) of environmental DNA extracted from 200 mLs of water on a single filter (dark grey columns) and from 800 mLs of water on 4 filters (light grey columns) with standard error bars.

We compared eDNA yields from 200 mLs of water on a single filter and 800 mLs of water across four filters and found an average eDNA concentration of 1.4 copies/µL (range: 0.76–2.31) for the single filter isolates and of 6.39 copies/µL (range: 4.87–8.39) for the four-filter isolates (Fig. 1). This resulted in an average of 4.4 times more copies/µL recovered in the four-filter isolation protocol relative to the single-filter (Wilcoxon: n = 6, V = 0, p < 0.05) at elution volumes of 100 µL. Of note, this procedure has been tested and works well on up to five filters without clogging (data not shown). Across all samples, positive and negative controls, we excluded two replicates with < 10,000 accepted droplets, which resulted in an average of 18,844 digital PCR droplets per well (N = 62, SE = 196), and a maximum of 21,056 droplets in a single well.

### Which combination of storage buffer and isolation method best reduces PCR inhibition from environmental samples?

**Figure 2:**
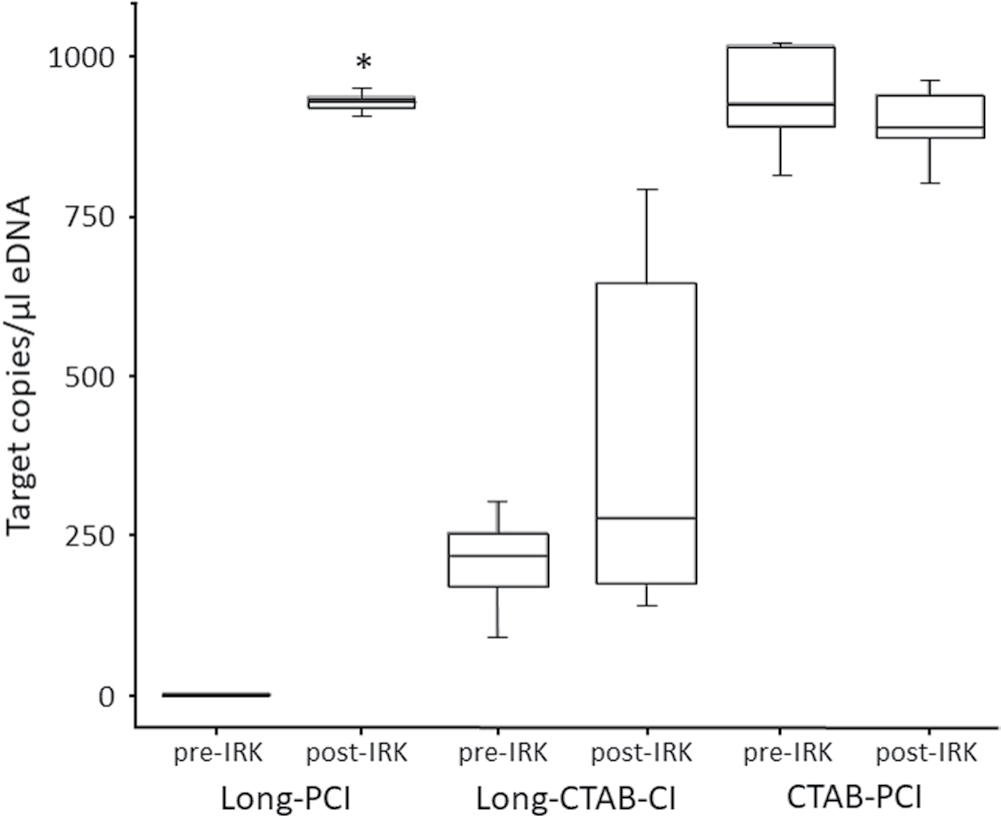
Box plots of average concentration of target gene copies (copies/µl) across the three storage and isolation treatments. (**A**) Long-PCI, Longmire’s storage buffer and phenol:choroform:isoamyl alcohol isolation, (**B**) Long-CTAB-CI, Longmire’s storage buffer and CTAB isolation and choroform:isoamyl alcohol cleanup, and (**C**) CTAB-PCI, CTAB storage buffer and PCI isolation. Asterisks(*) represent significant differences between the pre- and post-IRK treatment.

In a comparison of storage and isolation buffers, the CTAB-PCI method yielded the highest concentration of target gene copies (p < 0.05) of the three treatments (Table 1), and thus the most PCR inhibition removal prior to the IRK (Fig. 2). Interestingly, the IRK step did not significantly change the CTAB-PCI concentration (p > 0.05). However, considerable inhibition was observed in the Long-PCI treatment prior to IRK with a large increase in measured concentration of target gene copies post-IRK (p < 0.05). However, the post-IRK value of the Long-PCI was comparable to the CTAB-PCI method, both pre- and post-IRK. The Long-CTAB-PCI treatment yielded the least eDNA post-IRK. Across all samples, positive controls, standards, and negative controls, we achieved an average of 18,197 droplets per well (N = 187, maximum = 21,054, SE = 93) across two plates, with only one PCR replicate removed with droplets below 10,000. The pre- and post-IRK droplet counts were not found to be significantly different (paired T-test, p > 0.05).

**Table 1:** Average concentration of target gene copies (copies/µl) across the three storage and isolation treatments, pre- and post-Inhibitor Removal Kit (IRK).

Treatment	Preservation buffer	eDNA Isolation	eDNA copies/µl pre-/post-IRK
A. Long-PCI	Longmire’s	PCI	0.6/927.8*
B. Long-CTAB-CI	Longmire’s	CTAB-CI	206.6/406.3
C. CTAB-PCI	CTAB	PCI	933.7/892.9

(A) Long-PCI, Longmire’s storage buffer and phenol:choroform:isoamyl alcohol isolation, (B) Long-CTAB-CI, Longmire’s storage buffer and CTAB isolation and choroform:isoamyl alcohol cleanup, and (C) CTAB-PCI, CTAB storage buffer and PCI isolation. The Long-PCI treatment resulted in a significant increase (*) between the pre- and post-IRK treatment.

### Do genomic DNA concentration measurements increase in a linear fashion with increasing filtered amounts?

**Figure 3:**
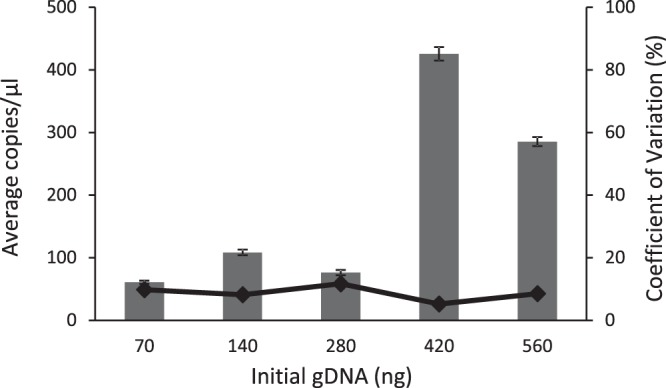
Average gDNA isolate concentrations (copies/µl) quantified by the droplet digital PCR with standard error bars for each of the five initial genomic DNA quantities (ng). The trendline represents the coefficient of variation (%) for each treatment.

To assess limitations on the application of genomic DNA (gDNA) during eDNA methods development, we filtered five increasing (doubling) amounts of grass carp gDNA using a single-filter protocol prior to ddPCR, ranging from 70 to 526 total ng (Fig. 3). We found a significant difference (p < 0.05) in measured target gene concentrations between the lower (70, 140, and 280 ng) and higher (420 and 560 ng) groups of initial gDNA quantities. The coefficient of variation remained low and invariable across the samples indicating high precision within and among the 25 PCR replicates (Fig. 3). Across all samples, and positive and negative controls (N = 141), we achieved an average of 13,697, and a maximum of 17,430 droplets per well (SE = 143). One replicate was removed as outlying data.

## Discussion

The low eDNA concentrations used for detection of invasive and imperiled species requires the careful optimization of eDNA isolation, inhibitor removal, and detection protocols for unique water types. The process of identifying these optimized adjustments to our protocols may include minor changes but can yield significant results worth sharing with the eDNA community. Among these studies, we found that increasing the volume of filtered water and using CTAB as a short-term (5–8 days) storage lysis buffer improved the yield of eDNA during isolation, reduced inhibition, and resulted in high precision of replicate measurements using ddPCR. We assessed our three protocols using Phenol:chloroform:isoamyl alcohol (PCI) DNA isolations, as they are low cost and in some cases have been shown to have increased yields of eDNA compared to commercial kits^22^. However, some of our findings also apply to isolation methods using commercial extraction kits.

Typically, filtering larger volumes of water accumulates more eDNA, which provides more accurate occupancy estimates^6^, however, this often also results in clogged filters. In our laboratory, with the regional waters we work with, we are typically able to filter ~200 mLs before the filters clog (depending on the particulate and microbiota loads). In Experiment 1, we scaled-up the isolation procedure to combine multiple filters, which effectively improved the expected eDNA yield by 10% in this experiment. Alternatively, multiple filters can be isolated separately and then combined during the final isolation step^37^, but previous testing of that process in our lab led to an overall loss of eDNA. Presumably this was due to the generally low concentration of eDNA on individual filters and the impact of even small amounts of loss during transfer between tubes at the combination step (data not shown). When working with very low copy numbers, loss is likely minimized when DNA “clumps” together. Therefore, a sufficient starting quantity of eDNA is necessary for detection post-processing. It may be important to meet or exceed this quantity by increasing filtered sample water volumes for optimal eDNA isolation, as indicated in Experiment 3, as DNA may bind to containers or the filtering apparatus. To account for the stochastic properties of source water containing only minute amounts of eDNA and to improve the precision of detection estimates, the filtration of larger water volumes may be beneficial for increased eDNA yield depending on the experimental system^15,23,25,41,42^. In addition to these benefits of being able to filter larger volumes of water, isolation of eDNA from all filters at once appears to improve the precision of the estimated detection probability^6^.

Using this protocol, we are able to distribute the liter of water among the individual filters in a manner that ensured the entire sample volume passed through the filters without clogging, enabling the isolation of all eDNA in the liter of water. We routinely isolate between two and five filters collectively, depending on particulate load, to allow for rapid filtration of ~1 L of water^43^. This volume ultimately depends on the microbiota level and particulate load present in the water samples and can change between collection sites. Alternatively, filters with larger pore sizes could be implemented to reduce clogging and allow more water to be filtered^20,24,44^. Although, we have observed that even with these larger pore sizes, eDNA may have been lost though the filter and filters frequently clogged with certain environmental samples (unpublished data).

In our second experiment, we tested three combinations of DNA storage and isolation methods within 5–8 days after filtering and found that the enhanced CTAB-PCI method resulted in the most eDNA yield and/or removal of inhibitors, prior to the IRK. Use of inhibitor removal kit columns are time consuming and can quickly become costly (~$1/sample) when multiple columns are needed for a thorough clean-up of highly inhibited samples. To augment the CTAB, PVP and β-mercaptoethanol were added to break down organic inhibitors like tannins and phenolics, as is often done in plant tissue DNA isolations^45,46^. It appears that the CTAB-PCI method removed much of the inhibitory compounds, as no significant change in concentration was found before and after the use of an IRK. The Long-PCI approach with an IRK resulted in a similar concentration as the CTAB-PCI method (with or without IRK). It is possible that the IRK removed inhibitory compounds in the Longmire’s buffer. However, due to the additional cost associated with the IRK step, and the opportunity for minor DNA loss, the CTAB-PCI method was selected as the optimal approach for isolation of eDNA from our water samples. The Long-PCI eDNA concentrations changed dramatically before and after the IRK, highlighting the effectiveness of the IRK in removing PCR inhibitors and/or the limited capabilities that PCI has for inhibitor removal. The Long-CTAB-CI method performed similarly before and after the IRK, potentially indicating some negative synergistic effect between the chemical solutions. Because this method was tested only on our local creek water, the applicability to other types or combinations of inhibitors and water samples may vary.

Renshaw, *et al*.^22^ found the Longmire’s lysis buffer to have improved eDNA yields as compared to CTAB during preservation of filters at room temperature for up to two weeks. This time frame facilitates field work and allows for extended laboratory processing time for eDNA isolation after filtration. However, due to the large volume of water needed for measurable eDNA yields, our laboratory typically freezes the water and then isolates after filtering within 5–8 days. Our data indicate that enhanced CTAB buffer had superior inhibitor removal properties, while Longmire’s buffer may be better suited for longer-term storage. We did perform a test by spiking gblock DNA into ultrapure water (without inhibitors) as a control for the creek water analysis; however, the concentrations of recovered target gene copies were ubiquitously low (data not shown). This may be a result of cell-free DNA being over-digested by the chemicals and/or not precipitating and supports the addition of carrier DNA to improve isolation of low quantities of eDNA.

In our final experiment, the expected linear relationship between starting and recovered target gene copies of increasing concentrations was not observed. Rather, we observed a significant increase in concentration between the lower three and upper two sample groups, specifically between the 280 ng and 420 ng gDNA samples (Fig. 3). High precision among replicates was indicated by a consistent and low coefficient of variation, suggesting that the assay performance did not influence this observation. This finding could be useful as a rough guide during methods development for judging whether more water should be filtered per sample to obtain accurate detection estimates of eDNA samples. Of note, the 280 ng measurement was lower than expected, potentially due to pipetting or dilution error. This study was conducted prior to the knowledge that eDNA filtration is primarily collecting cell-bound eDNA and also the evidence of eDNA binding to plastics; although some labs still develop protocols using cell-free eDNA^20^. We found evidence that low quantities of filtered eDNA may not result in the expected linear trend as concentrations increase. These findings can be used during methods development to inform the amount of cell-free eDNA that is required to effectively precipitate DNA in PCI isolations. We suggest that low retention plastics (except while pelleting DNA) should be used to reduce of eDNA loss. Further, it would be beneficial to investigate the use of carrier DNA, which binds to and helps pellet free-floating eDNA in a sample, especially during optimization and testing of protocols prior to the use of experimental water^14,38–40,47^. Learning from this experiment, we used environmental creek water in our first and second experiments, as opposed to purified lab water, to incorporate natural DNA carriers found in the creek water. Further, use of a synthetic gene in the assay is recommended for accurate quantification of initial DNA concentrations. Improvements in accuracy and consistency in eDNA detection are central to management decisions for imperiled and invasive species. As identified here, sustained optimization and development of methods are needed as the field advances and expands.

## Methods

### General methods used throughout

All analyses utilized sterile technique and clean rooms to separate the experimental steps. Low-retention filter pipette tips were used for all eDNA laboratory processing. Grass Carp tissue from fin clips were isolated for use in our eDNA studies following standard protocols using Qiagen’s DNeasy Blood and Tissue kit (Valencia, CA, USA). These tissue DNA samples were used throughout the study in cell-free DNA spiked samples and as positive controls.

#### Water filtration and DNA preservation

All water samples were collected and/or stored in 1 L polypropylene grab bottles. For all experiments, we used a standard duty dry piston pump (Welch model 2522B-01, Niles, IL, USA), at approximately 26 cm Hg vacuum, to filter water through a polyethersulfone filter (PES; 0.45 micron, 47 mm) from Sterlitech (Kent, WA, USA) placed on a 47 mm magnetic filter funnel (Pall, Port Washington, NY, USA). Filters were placed in microcentrifuge tubes containing lysis buffer (see individual Experiments for details) to preserve the DNA, then stored at 4 °C until DNA isolation was performed, within 5–8 days.

#### Phenol-chloroform-isoamyl alcohol DNA isolation

Water samples were isolated following one of three methods: the modified PCI protocol from Renshaw, *et al*.^22^, our scaled-up protocol (described in Experiment 1), or a CTAB isolation protocol (described in Experiment 2(B)). Briefly, Renshaw, *et al*.^22^ incubated the microcentrifuge tubes containing the filters and lysis buffer for 10 min in a 65 °C water bath. The tubes were then allowed to cool to room temperature for up to 10 min. Once cool, 900 µL PCI (25:24:1; Amresco, Solon, OH, USA) was added to the tubes, vortexed until the PES filter fully disintegrated, and centrifuged at 13,200 rpm for 5 minutes using an Eppendorf 5415D tabletop microcentrifuge (Eppendorf North America, Hauppauge, NY). The upper aqueous layer was transferred to a new tube, leaving a millimeter of the aqueous layer to prevent carryover of the undesired lower organic layer. The same procedure was then followed using 700 µL of chloroform:isoamyl alcohol (CI; 24:1; Amresco, Solon, OH, USA). After transferring the upper aqueous layer to a new tube, 1 mL of ice cold 95% ethanol and 20 µL of 5 M NaCl were added and the solution was allowed to precipitate overnight at −20 °C. The next day, the tubes were centrifuged at 13,200 rpm for 10 minutes and the ethanol mixture was removed with a pipette. The pellets were washed with 1 mL of cold 70% EtOH, re-centrifuged for 5 minutes, and the liquid was pipetted off. The tubes were allowed to air dry in a fume hood at room temperature and the pellets were rehydrated with 100 µL (Experiments 1 & 2) or 50 µL (Experiment 3) low EDTA 1X Tris-EDTA (TE) buffer for 30+ minutes. All DNA and eDNA concentrations were measured using an Epoch spectrophotometer (BioTek, Winooski, VT, USA) and the samples were stored in 1.5 mL microcentrifuge tubes at −80 °C.

#### PCR assays

For all experiments, ddPCR reaction mix and cycling conditions followed previously published protocols (see^48^). The assay included the forward GC‐CB‐eDNA‐7‐F (5′‐CAA CGA CGC GCT AGT CGA T‐3′) and reverse primers GC‐CB‐eDNA‐7‐R (5′‐TCC AAA GTT TCA TCA TGC AGA GA‐3′), and a TaqMan probe GC‐CB‐eDNA‐A‐7‐Prb (5′‐TTCCCACACCATCTAA‐3′) with VIC (Experiments 1 & 2) or 6‐FAM (Experiment 3) and an NFQ‐MGB quencher. The probe label was held constant within an experiment. In our droplet digital PCR assays, we included an internal positive control (IPC) PrimePCR Probe assay containing 0.3125 µl or 1 µl PreAmp and 0.20 µl of diluted template (YWHAZ, Rhesus Monkey; BioRad) in all PCR assays to distinguish between true target negatives and PCR inhibition. Droplet digital PCR data were analyzed using QuantaSoft Version 1.7.4.0917 software (BioRad, Hercules, CA, USA). Following ddPCR amplification, the droplets were analyzed using the QX200 Droplet Reader (BioRad, Hercules, CA, USA). Thresholds were manually set in the software programs in accordance with manufacturers recommendations.

#### Data analysis

Droplet digital PCR wells that contained less than 10,000 total droplets were removed from downstream analysis. Statistical analysis was performed using R^49^ or Microsoft Excel, 2010 (Microsoft, Redmond, WA). The concentration of target gene copies in the well was calculated using ednaoccupancy in R^50^. Sample concentrations lower than the controls were below the limit of quantification and were removed. The metrics reported for the ddPCR assay include average and maximum values, and the associated standard error of the accepted droplets across all reactions. Average “target.estimate” values were determined for the positive PCR replicates for each sample^50^. These target estimates were corrected to report copies of target eDNA per microliter. Boxplots were produced to visualize the data using the ‘ggPlot’ package^51^ in RStudio^52^. Outliers were identified using the ‘outliers’ package^53^ in RStudio and removed from final analysis. Statistical comparisons between groups were performed using the ‘wilcox.text’ analysis in the ‘stats’ package in R^49^.

## Experimental Methods

### Do multiple filter isolations result in greater eDNA yield?

#### Environmental DNA filtration

We compared eDNA yields from the isolation of a single filter (200 mL water) vs multiple filters (4 filters and 800 mL of water total [200 mL each filter]) to assess the filtration of larger volumes of water while minimizing the filter clogging. We collected six samples of ~950 ml pond water from a local aquaculture farm pond (Hampton, FL) containing live Grass Carp and added 34 ml of a DNA preservative (1 mL of sodium acetate (3 M) and 33 mL of 95% ethanol) before capping and gently mixing by rotating the bottle 4–5 times^54,55^. One negative field control sample was also prepared at the site by pouring purified water into a bottle and adding preservative. The bottles were then transported on ice to the lab and stored at 4 °C overnight for filtering the following day. The bottles were gently mixed by inverting several times and filtered through a 1.2 micron PES filter (described in general methods). From each liter of pond water, a 200 mL volume was filtered through a single filter and the filter was then placed in buffer (described in next paragraph) in a 2 mL microcentrifuge tube. The remaining 800 mL was filtered through four PES filters (~200 mL/filter), and all four filters were then combined into a single 5 mL microcentrifuge tube with buffer. Filtering controls using ultrapure water were analyzed to check for contamination.

The filters were stored in an enhanced Cetyl trimethylammonium bromide (CTAB) lysis buffer (EDTA 0.5 M [0.04 mL/mL], Tris 1 M [0.1 mL/mL], NaCl 5 M [0.28 mL/mL], PCR H_2_O [to 1 mL], CTAB [0.02 g/ml], PVP [0.04 g/mL], β-mercaptoethanol [5 μL/ml]) made up the day of use. The CTAB buffer included the addition of PVP (polyvinylpyrrolidone) and β-mercaptoethanol to break down organic material such as tannins and phenolics^56^. A volume of 0.9 mL and 1.7 mL of the CTAB buffer was added to the 2 mL and 5 mL tubes, respectively, and the filters were stored at 4 °C for 5–8 days before DNA isolation.

#### DNA extraction and analysis

We utilized the standard PCI protocol to isolate the single filter in the 2 mL tubes (see general methods). To process the 5 mL tubes containing four filters, we modified our standard protocol by increasing the volumes of reagents: 1.7 mL PCI, 1.5 mL CI, 48 μL 5 M NaCl and up to 3 mL ice-cold 95% EtOH. To accommodate the 5 mL tubes, a large centrifuge (Sorvall XTR benchtop centrifuge [Thermo Scientific, Waltham, MA]) was used at 10,000 rpm for all centrifugation steps. The eDNA concentrations were measured using an Epoch spectrophotometer (BioTek, Winooski, VT, USA) and the samples were stored in 1.5 mL microcentrifuge tubes at −80 °C. All samples were analyzed using ddPCR described in the general methods. The number of target gene copies detected from the single filter and four-filter extractions were compared using a paired Wilcoxon Signed-Rank analysis to test for significance. It should be noted that this process has been successfully completed using up to five filters in our laboratory (data not shown).

### Which combination of storage buffer and isolation method best reduces PCR inhibition from environmental samples?

#### Water sample collection

To assess which storage buffer and isolation procedure best reduces the effects of PCR inhibitors, we tested three different combinations of storage and isolation protocols. The three protocols were (A) Long-PCI: Longmire’s storage buffer with PCI isolation, (B) Long-CTAB-CI: Longmire’s storage buffer with CTAB isolation and CI cleanup, or (C) CTAB-PCI: CTAB storage buffer with PCI isolation (see Table 1). To test the effects of inhibitors on these methods, tannin rich, tea-colored water was collected from a local creek (Gainesville, FL). The water was carefully mixed to ensure even distribution of compounds and then subdivided into sterile 1 L bottles for transportation. It was stored at 4 °C until filtration.

#### Filtration and eDNA preservation

Preliminary assessment of creek water samples indicated that there were portions of the creek which contained high concentrations of inhibitors, and areas with lower inhibitor concentrations. Therefore, 200 mL of creek water with high concentrations of inhibitors was mixed with 200 mL of creek water with lower concentrations to create experimental samples containing moderate levels of inhibitors. Each 400 mL sample was filtered through a single filter using our standard methods with five replicate water samples filtered for each of the three protocols (N = 15 total filters). The filters were then individually placed in 5 mL microcentrifuge tubes containing 2 mL of either enhanced CTAB storage buffer (described in Experiment 1) or Longmire’s Buffer (stock: 5 mL Tris (1 M), 10 mL EDTA (0.5 M), 2.5 mL SDS (10%), 0.1 mL NaCl (5 M), and 32.4 mL DNA-free H2O)^57^. Tubes were stored at 4 °C until isolation within 5–8 days. Immediately before isolation, the tubes were spiked with 100 μL of gBlock (100 copies/µL; Integrated DNA Technologies, Coralville, IA, USA) containing the Grass Carp synthetic gene sequence^48^.

Protocols (A) and (C) followed the 5 mL PCI isolation specified in Experiment 1. Protocol (B) utilized a CTAB-CI isolation protocol modified from Palomec and Mccauley^56^. Briefly, the 5 mL tubes with the filters and Longmire’s buffer were placed in a 65 °C water bath for 10 minutes. After cooling to room temperature, 500 μL of the same enhanced CTAB buffer used in Experiment 1 were added and the tubes were vortexed for 10 seconds. The tubes were then incubated in a 55 °C water bath for 1 hour. After incubation, 500 μL of CI (24:1 Amresco) were added to each tube. The tubes were then cooled at room temperature for 10 minutes and vortexed for 10 seconds to allow the CI to fully break down the PES filters. The tubes were centrifuged at 10,000 rpm for 10 minutes and the aqueous layer was transferred into a new 5 mL microcentrifuge tube. Then 48 μL of 5 M NaCl and up to 3 mL ice-cold 95% EtOH were added to each tube and the DNA was precipitated at −20 °C overnight. The samples were pelleted and rehydrated following the 5 mL protocol specified in Experiment 1.

#### Digital droplet PCR and statistical analysis

Four PCR replicates of each of the 15 isolated samples were analyzed by ddPCR (see general methods for details) before and after performing one inhibitor clean-up using the commercially available OneStep PCR Inhibitor Removal Kit (IRK; Zymo Research, Irvine, CA, USA) following the manufacturers protocol (Table 1). The Grass Carp gBlock was used to produce six standards along a 1:4 dilution series ranging from 800 to 0.78 copies per reaction. A positive control of 0.1 ng/μL Grass Carp DNA extract from tissue was also run in quadruplicate, as were the standards and NTCs. Pre- and post-IRK concentrations of target gene copies were tested using a paired Wilcoxon Signed-Rank analysis, as well as an un-paired assessment between treatment types. A boxplot was used to visualize the pre- and post-IRK DNA concentrations (Fig. 3).

### Do genomic DNA concentration measurements increase in a linear fashion with increasing filtered amounts?

#### Water filtration and DNA isolation

We assessed five quantities of genomic Grass Carp DNA between 70 and 560 ng spiked into ultrapure water prior to filtration (on a single filter), replicated five times per quantity. After filtration, the filter was placed in a 2 mL microcentrifuge tube and completely immersed in 900 µLs of Longmire’s buffer (described in Experiment 2). We then followed our standard PCI isolation protocol (see general methods) to extract the eDNA from the filters.

#### Droplet Digital PCR and statistical analysis

The ddPCR was performed according to the methods described in the general methods section above. The five eDNA quantities were each assessed across five PCR replicates (in 25 total PCRs) on two plates. On the two experimental plates, a grass carp tissue positive control (N = 2 and 5) and no template controls (NTCs; N = 2 and 10) were included. The replicate concentration values for ddPCR analyses were calculated and outliers were removed as outlined in the general methods. Significant differences between the groups were assessed using a Grubb’s test. The average copies/µl were then reported for the five different treatments along with their standard error and the coefficient of variation.

### Disclaimer

Any use of trade, product, or firm names is for descriptive purposes only and does not imply endorsement by the U.S. Government.
